# Supplementary material for: An individual’s physique is associated with the length of the reconstruction route via the retrosternal approach
Source: PLoS One. 2023 Mar 31;18(3):e0283890. doi: 10.1371/journal.pone.0283890 (PMC10065263; doi:10.1371/journal.pone.0283890)
Supplement: Attachment — Submitted filename: Response to Reviewers.docx [file pone.0283890.s004.docx]

**Responses to Editor’s and Reviewers’ Comments**

**To Editor**

**Editor’s comment**

Your study is observational and therefore causality cannot be inferred. Please update your title to reflect this (replace ‘affects’ with ‘is associated with’) and check throughout the manuscript for similar instances (I found examples in both the abstract and conclusions).

**Response to comment**

Thank you for your kind suggestion. We have checked throughout the manuscript for similar instances. We have replaced “affect” with “is associated with” in the title and body of the manuscript according to your suggestion (Page 1, lines 1 & Page 1, lines 15 & Page 2, lines 18 & Page 2, lines 21 & Page 3, lines 21 & Page 5, lines 5).

**To Reviewer #3**

**Reviewer’s comment 1**

The authors concluded that choosing the posterior mediastinum route reduces anastomosis-related complications in obese patients with long distance of the retrosternal reconstruction route. However, the relationship between the length of the reconstruction route and the incidence of anastomotic leakage was not shown in this paper.

**Response to comment 1**

Thank you for your pertinent comment. In the RS reconstructions (n=40), the RS ≥ ES group had 30 cases and the RS < ES group had 10 cases; anastomosis-related complications occurred in six cases and one case in the two groups, respectively, which were not significantly different (*p*=0.66). Our series did not reveal a relationship between the length of the reconstruction route and anastomotic leakage because of the small number of cases. Furthermore, five overweight cases had BMIs >25 kg/m^2^; in all these cases, the RS route was longer than the PM route, and no anastomosis-related complications were observed. Our study revealed no relationship between BMI and anastomotic leakage; however, the study findings revealed that the reconstruction length was related to the individual’s physique. Further studies exploring the relationships between reconstruction lengths, individual’s physique, and outcomes of esophagectomies are needed to confirm these findings. According to your suggestion, we have added the following sentence to the Results and Discussion sections: “The RS ≥ ES group included 30 cases, whereas the RS < ES group included 10 cases; meanwhile, the anastomosis-related complications in each group comprised six cases and one case, respectively, in which there was no significant difference (*p*=0.66)” (Page 10, lines 7-10). “Although our series did not reveal a relationship between the length of the reconstruction route and anastomotic leakage and did not reveal a relationship between the BMI and anastomotic leakage” (Page 16, lines 6-7).

**Reviewer’s comment 2**

As for the similar question, is there a difference in the incidence of suture failure between the RS ≥ ES group and the RS < ES group?

**Response to comment 2**

Thank you for your comment. As noted in the response to comment 1, there was no difference in the incidence of suture failure between the RS ≥ ES and RS < ES groups. According to your suggestion, we have added the following sentence to the Results section: “The RS ≥ ES group included 30 cases, whereas the RS < ES group included 10 cases; meanwhile, the anastomosis-related complications in each group comprised six cases and one case, respectively, in which there was no significant difference (*p*=0.66)” (Page 10, lines 7-10).

**Reviewer’s comment 3**

Please add the references of Collard and triangulating stapled anastomosis. (line 79-80)

**Response to comment 3**

Thank you for your advice. We have added the following references for Collard and triangulating stapled anastomosis according to your suggestion:

22. Collard JM, Romagnoli R, Goncette L, Otte JB, Kestens PJ. Terminalized semimechanical side-to-side suture technique for cervical esophagogastrostomy. Ann Thorac Surg. 1998;65: 814-817.

23. Orringer MB, Marshall B, Iannettoni MD. Eliminating the cervical esophagogastric anastomotic leak with a side‐to‐side stapled anastomosis. J Thorac Cardiovasc Surg. 2000;119: 277-288.

24. Furukawa Y, Hanyu N, Hirai K, Ushigome T, Kawasaki N, Toyama Y, et al. Usefulness of automatic triangular anastomosis for esophageal cancer surgery using a linear stapler (TA-30). Ann Thorac Cardiovasc Surg. 2005;11: 80-86.
